# Complete Genome Sequence of the Carotenoid-Producing Strain Gordonia ajoucoccus A2

**DOI:** 10.1128/MRA.00662-20

**Published:** 2020-09-10

**Authors:** Soon Jae Kwon, Yong Jin Choi, Ju Min Kim, Pyung Cheon Lee

**Affiliations:** a Department of Molecular Science and Technology, Ajou University, Woncheon-dong, Yeongtong-gu, Suwon, South Korea; b Department of Applied Chemistry and Biological Engineering, Ajou University, Woncheon-dong, Yeongtong-gu,Suwon, South Korea; c Department of Energy Systems Research, Ajou University, Suwon, South Korea; d Department of Chemical Engineering, Ajou University, Suwon, South Korea; University of Maryland School of Medicine

## Abstract

Gordonia ajoucoccus strain A2, isolated from crude oil-contaminated soils, synthesizes yellow keto-γ-carotene from various *n*-alkanes as the sole carbon source. Its complete genome sequence consists of a single circular chromosome (5,090,254 bp, 67.3% G+C content). Seven putative genes were identified supporting the proposed keto-γ-carotene pathway of *G. ajoucoccus* A2.

## ANNOUNCEMENT

Previously (1), we isolated and characterized a new strain, Gordonia ajoucoccus A2, which can degrade *n*-alkanes of variable chain lengths from oil-contaminated soil. *G. ajoucoccus* A2 produced carotenoids when grown in media containing *n*-alkanes as the sole carbon sources. γ-Carotene and keto-γ-carotene are the main carotenoids in *G. ajoucoccus* A2 (1). Carotenoids are widely used as cosmetic ingredients, antioxidants, and food or feed additives (2–4). Studying *G. ajoucoccus* A2—including whole-genome sequencing—to validate it as a carotenoid producer is worthwhile. These genomic data can form the basis for elucidation of carotenoid biosynthesis mechanisms and for metabolic engineering of *Gordonia* strains (5–8) suitable for large-scale fermentative production of other biotechnologically important carotenoids from industrial discharge or other sources of *n*-alkanes.

The strain was isolated from a local Korean chemical company (Seok-Rim Chemicals) and aerobically cultured at 30°C in a 125-ml serum bottle containing 20 ml of mineral salt medium (1) with 0.5% (wt/vol) fructose as the sole carbon source for 3 days. Genomic DNA was extracted via a genomic DNA kit (Macrogen, South Korea) with RNase A treatment and sequenced in single-molecule real-time (SMRT) sequencing cells using PacBio RS II SMRT technology (Pacific Biosciences, CA) on the HiSeq 2000 platform (Illumina, USA). Both procedures were performed by DNA Link, Inc. (Seoul, South Korea). The sequencing libraries were prepared using the SMRTbell template prep kit 1.0 (PacBio RS II) and the TruSeq Nano DNA kit (Illumina). All software was run with default settings unless stated otherwise. After subread filtering of the PacBio raw data, 141,490 long reads with an average length of 11,140 bp (total, 1,130,407,021 bp; genome coverage, >220-fold; mean read score, 0.829) were generated and *de novo* assembled in the Canu v1.3 (9) assembler with the parameter genomeSize=5m. The overlapping regions at both ends of one contig were trimmed to create unique stretches on both ends using Circlator (10) (b2r_length_cutoff=60000, 100000, or 200000). The resulting one contig was error corrected in Quiver (11) for three cycles. The error-corrected assembly was further polished using Pilon v1.22 (--fix bases) (12) with trimmed paired-end reads (total, 8,082,491 reads; genome coverage, >126-fold), which were obtained from 2 × 251-bp paired-end reads (8,427,944 reads totaling 2,115,400,630 bp) in Sickle v1.33 (https://github.com/najoshi/sickle). The assembly statistics were calculated using stats.sh from BBmap v38.68 (https://sourceforge.net/projects/bbmap/). Genome annotation and gene prediction were conducted using the Prokaryotic Genome Annotation Pipeline (PGAP) (13).

The genome consists of a single 5,090,254-bp circular chromosome with 67.3% G+C content. Annotation revealed 4,825 coding DNA sequences and 110 encoded RNAs (9 rRNAs and 47 tRNAs). Seven genes for keto-γ-carotene biosynthesis were predicted in the genome, namely, one gene encoding geranylgeranyl diphosphate synthase (CrtE), one encoding phytoene synthase (CrtB), two encoding phytoene dehydrogenase (CrtI), one encoding lycopene γ-cyclase (CrtL), and two genes encoding γ-carotene ketolase (CrtO).

### Data availability.

This whole-genome shotgun project has been deposited in DDBJ/EMBL/GenBank under the accession number CP052884.1. The SRA/DRA/ERA accession numbers are SRR11612832 (PacBio) and SRR11612831 (Illumina). The BioSample and BioProject numbers are SAMN14731745 and PRJNA628123, respectively.
